# Obstetrics, and Diseases of Women and Children

**Published:** 1861-11

**Authors:** Wm. B. Atkinson

**Affiliations:** Lecturer on Obstetrics, and Diseases of Women and Children; Physician to the Department of Obstetrics and Diseases of Females of the Howard Hospital and Infirmary for Incurables, Philadelphia


### OBSTETRICS, AND DISEASES OF WOMEN AND CHILDREN.

BY WM. B. ATKINSON, M.D.,

LECTURER ON OBSTETRICS, AND DISEASES OF WOMEN AND CHILDREN; PHYSICIAN TO THE DEPARTMENT OF OBSTETRICS AND DISEASES OF FEMALES OF THE HOWARD HOSPITAL AND INFIRMARY FOR INCURABLES, PHILADELPHIA.

### I.—*The Timely Use of the Obstetric Forceps.* By EDWARD B. SINCLAIR, M.D., Dublin.

The chief object of the obstetric practitioner should be the preservation of the structures of both mother and child, and the prevention of fever and inflammation during and after parturition.

The early application of the forceps prevents inflammation of the mother's tissues, and its results. The free and timely use of this instrument is a powerful means of warding off disease and death, especially in public obstetric institutions.

Out of 13,748 deliveries at the Dublin Lying-in Hospital, (225 by the forceps,) there occurred 20 cases of sloughing of the soft parts; 7 of these were in deformed pelvis, after perforation and crotchet delivery; 5 in tedious labors, not instrumental; 2 in prolapse of the funis, and 1 in retained placenta; in neither of which the forceps was used, leaving but 5 cases of sloughing after the use of the forceps. There were only four examples of fistula after convalescence: 2 completely closed; 1 contracted to a minute opening; and in 1, the woman subsequently died of peritonitis. Had the forceps been used more freely and earlier, Dr. Sinclair is almost certain that the 5 cases which occurred in tedious labors, without instruments, and the 5 with the forceps, would not have been recorded.

In public institutions there is a strong disposition to puerperal diseases. Lessen the tedious cases and this will not occur. Thus, in 242 deliveries in one period, the forceps never used, there were 17 deaths, not to mention cases of disease. In 3886 deliveries in another period, there were 70 deaths, and the forceps was used 38 times. In 9620 deliveries in a third period, there were 78 deaths from all causes, and the forceps was used 187 times.

In the above-mentioned institution the rules finally adopted were, never to wait for positively bad symptoms. In doubtful cases, and where the application was not difficult, the forceps were employed; or if the fœtal heart seemed about to fail after ergot, or in slight narrowing of the pelvis, and also in every case where it is certainly seen that bad symptoms will arise, and that the woman will not previously deliver herself, the rule was to apply the forceps as soon as justifiable.—(*Dublin Jour. Med. Sci.*, August, 1861.)

II.—*Neuralgia after Parturition.* By HENRY NUTTALL, M.D., Leicester, England.

Of this complication of the puerperal state, but little mention is made by the authorities in obstetrics, though it would appear of sufficiently frequent occurrence. Dr. Nuttall has recently encountered two cases, one a tedious labor, and the other remarkably easy. In both, the pain was felt in one of the lower extremities, extending from the foot up to the hip. The treatment, consisting of opiates, steel, and quinine, was but partially successful, time acting greatly in the cure. —(*Brit. Med. Jour.*, July 27th, 1861.)

[We have recently met with three cases of neuralgia immediately succeeding parturition; in one the pain was confined to the breast, was constant and agonizing. In the others, it was more general, and changing constantly from one part to another. The treatment which relieved the pain, consisted of the administration of quinia, belladonna, and morphia, in full doses.—W. B. A.]

III—*Unusual Elongation of the Fœtal Head as a Cause of Difficulty in the Application of the Ordinary Obstetric Forceps.* By GRAILY HEWITT, M.D., London.

The author's attention has been directed to the subject by the fact that, in a particular case, a difficulty was experienced in applying the short forceps. After the labor had been completed, the head of the child, compressed and moulded to the shape of a narrow and unyielding parturient canal, was found to be unusually elongated in the occipito-mental diameter, measuring six inches half an hour after birth. The actual size of the head was not remarkable. The difficulty with the forceps was due to the fact that the side of the head presented, owing to its elongation, a curve different from that ordinarily met with, and the blade of the instrument was not long enough, while, at the same time, it was too sharply curved to meet the requirements of the case. The only modification of the forceps hitherto made, had reference to the position of the head in the passage. The shape of the head should also be regarded. The author exhibited an instrument, with a blade of eight inches, the curve that of a circle of fourteen inches, and identical in other respects with the ordinary short forceps. Thus modified, he believed it would answer every indication. In the long, tedious labors of primiparæ, this instrument would come into requisition.—(*Med. Times and Gaz.*, June 15th, 1861.)

[Dr. Chas. P. Bethel, of this city, has produced a modification of the Davis forceps, which would seem likely to meet all such difficulties. In it, the curve of Carus is closely imitated, at the same time the blade is admirably fitted to the child's head, and by the length of the blade, it would be fully equal to the requirements of these exceptional cases alluded to by Dr. Hewitt.—W. B. A.]

IV.—*Is Ergot, when administered to the Mother during Labor, dangerous to the Life of the Child?* By R. UVEDALE WEST, M.D., Edinburgh.

In a previous paper, the author reported 69 cases in which he had administered ergot, out of 278 labors; 9 children were still-born, viz., 2 putrid at birth; 2 after labors with hemorrhage; 1 footling, with hydrocephalus and consequent fatal compression of the funis; 1 in which there was evidence of latent compression of the funis; 1 a difficult primiparous forceps delivery; 1 a vectis delivery, the mother seriously ill from excessive œdema; and 1 dead without any assignable cause. Since then he has attended 734 labors: ergot was given 172 times, including one case of twins, thus giving 173 children. Only 5 were still-born, viz., 3 putrid at birth; 1 with placenta prævia and profuse hemorrhage—premature; and 1 with prolapsed funis.

From these statistics, Dr. West believes there is no sufficient evidence to justify the doctrine that ergot is dangerous to the life of the child.

As to the mother, in these 1013 labors there were 7 deaths in the lying-in month, of which 1 had taken the ergot; 18 cases of incarcerated placenta, of which 5 had ergot; 25 of *post-partum* hemorrhage, 5 after ergot; 30 of puerperal disease, 9 after ergot. Hence he concluded this agent had no influence either in causing or preventing parturient accidents or diseases. Probably it might, in deficient uterine action, control hemorrhage and after-pains.

He considered it immaterial, in the use of ergot, whether the os was rigid or supple; the liquor amnii discharged or not; the mother a primipara or not. It was useful to improve uterine action, and only dangerous where such action would be, as an arm presentation with the membranes ruptured, as then turning would by it be rendered more difficult.

DR. GRAILY HEWITT thought the conclusions were fully borne out by the data, and the inactivity of ergot was proved.

DR. TYLER SMITH differed: Ramsbotham's statistics proved the reverse. Those of Hardy, McClintock, Johnstone, and Sinclair were so convincing that in the Dublin Lying-in Hospital it was a rule to use the forceps, unless the child was delivered within twenty minutes after giving ergot. Whether it acted as a poison or mechanically, he had no doubt of its deleterious effects. It should be regarded only as a *post-partum* remedy.

DR. J. BRAXTON HICKS felt sure that occasionally the effect of the remedy was to cause continuous contraction in the circular direction rather than longitudinally, whereby the child was so tightly clasped that it perished from pressure on the funis.

DR. BARNES regarded the toxical effects of ergot as established. He had observed direct injurious effects, as the rupture of the perineum by the violent driving of the child through the pelvis. After its administration, there was no means of controlling the action of the uterus.

DR. HALL DAVIS had seen one case, if not more, where rupture of the uterus was traceable to its use. He only considered it to act mechanically.

DR. CHOWNE agreed in this belief: the agent required caution in its use. It did harm by the continuous contraction induced.—(*Brit. Med. Jour.*, July 27, 1861.)

V.—1. *Placenta Prœvia.* By WILLIAM READ, M.D., Boston.

From a volume entitled "Placenta Prævia; its History and Treatment," by Dr. Read, we obtain the following summary:—

The danger to the mother increases as the period at which the labor comes on approaches the full term. It is therefore better to terminate the labor after it has really begun, as soon as compatible with the safety of the patient, than to endeavor to conduct the pregnancy to the full term.

The danger to the mother is less when the os uteri is completely covered than when a portion only is involved in the attachment of the placenta; and least of all, where the attachment becomes nearly or quite central with reference to the os. Under these circumstances, there is a strong probability, if the contractions are vigorous enough, that the placenta will be thrown off and expelled into the vagina, and the hemorrhage be checked.

The *condition* of the mother is more important in making the prognosis than the amount of blood lost. When the pains are vigorous and permanent, the head presenting, the os in good condition, and the strength not materially impaired, *rupturing the membranes,* by letting off the waters, and bringing down the head on the os, will generally place the mother in safety. But if there is a want of tonic power, or forced delivery be probable, this method will increase the difficulty of the operation, and the danger to the mother.

The danger to the mother is increased by artificial delivery. But this is owing not to the operation itself, so much as to the exhaustion at the time, and with a favorable condition of the mother this is reversed. Hence this operation should be performed before exhaustion comes on, and should not be delayed an instant beyond the time when the state of the os will permit the introduction of the hand. When, from any cause, artificial delivery is forbidden, the placenta should be wholly separated and the strength of the mother recruited by every means, till she can be delivered.

The tampon may be used where, with some flooding, the os remains rigid. But never when artificial delivery is possible.

Ergot should not be administered when there is a probable necessity of terminating the labor by an operation, unless at such an interval that the effect of it is either exhausted or will not come on until after the operation is finished, or the condition of the mother is such that it will merely act as a stimulant.

Where the exhaustion is excessive, and version is the only alternative, after the feet have been brought down, the body of the child should be left undelivered until the uterus has been roused to contract and a firm condensation of its walls has been secured; or, at least, it should be withdrawn so slowly as to prevent the evil consequences which sometimes follow too sudden delivery.

2.—*Ibid* By CHARLES CLAY, M.D., Manchester.

Dr. Clay has, for nearly forty years, put in practice Mr. Kinder Wood's plan of detachment of the placenta by means of the forefinger.

The statistics of version and immediate delivery are—

| | |
|---|---|
| Fatal to mother . . . . . . | 1 in 3 cases. |
| Fatal to child . . . . . . . . | 1 in 2 cases. |

From the best authorities, those of detachment and then leaving the case for nature, are—

| | |
|---|---|
| Fatal to mother . . . . . . . | 1 in 44 cases. |
| Fatal to child . . . . . . . . | 1 in 5 cases. |

In Dr. Clay's practice he has *never known it to fail.* The hemorrhage immediately ceases. He has never witnessed any bad consequences from this plan; there is less violence done, the danger is reduced, the results are far more favorable.—(*Glasgow Med. Jour.*, July, 1861.)

3.—*Ibid.* By ROBERT BARNES, M.D., London.

After reporting in detail 3 cases, Dr. Barnes sums up as follows :—

Detach with one finger, passed through the cervix, all that portion of the placenta adhering to the cervix. Tap the membranes and drain off the liquor amnii. If the uterus remains flaccid, and *hemorrhage continues*, accelerate the delivery by artificial aid, as, where the cervix is dilated, turn; where otherwise and rigid, employ the caoutchouc dilater.—(*Lancet*, June 1, 1861.)

4.—*Ibid.* By Thomas Radford, M.D., Manchester.

Dr. Radford, being desirous of obtaining statistics on the subject of the treatment of placenta prævia, solicits communications from practitioners as to the following particulars:—

The number of cases of this accident; partial or complete; state of the os; general condition of the patient; degree of hemorrhage; presentation of the child; mode of practice. Was it (*a*) to leave it to the natural powers, (*b*) to merely rupture the membranes and then trust to nature, (*c*) to turn and deliver, (*d*) to completely detach the placenta and then leave the child to be expelled by the uterus, (*e*) to completely detach the placenta, extract it, and then deliver? (*f*) was the placenta separated or detached by the hand or by a finger? (*g*) what was the degree of hemorrhage which occurred after the complete detachment of the placenta? (*h*) what was the mother's fate? (*i*) what was the child's fate? (*k*) was galvanism applied? (*l*) at what period of pregnancy, and if first, or which other pregnancy?—(*Med. Times and Gaz.*, Aug. 17, 1861.)

[Being desirous to aid Dr. Radford in his inquiry upon this subject, I would be happy to receive communications from American physicians, and, where desired, prepare them as Dr. R. requests, and forward them to him.—W. B. A.]

VI.—*On the Indications and Operations for the Induction of Premature Labor, and for the Acceleration of Labor.* By Robert Barnes, M.D., London.

The author traces the history of the various modes of inducing labor, and discusses the action and merits of each. The only method used for a long time was the puncture of the amniotic sac directly over the os uteri. Experience showing that the premature escape of the liquor amnii led to the frequent peril of the child from undue compression in being forced through an imperfectly dilated cervix, Hamilton substituted the separation of the membranes for a given space from the lower segment of the uterus; and Hopkins tapped the sac at a point remote from the os. Others gave ergot. Merrem and Krause placed a flexible catheter in the uterus, leaving it there to excite pains. Braun then spoke highly of this plan. In 1820 Brunninghausen led the way in a series of proceedings for dilating the cervix by plugs, etc. In 1842 Huter proposed to expand the vagina by inflating bladders introduced in a flaccid state. Braun then introduced his caoutchouc colpeurynter. These contrivances have been followed by inflammation of the genitals, and death. Galvanism, irritation of the breasts, and the carbonic acid gas douche were also used. The latter caused sudden death in the hands of Scanzoni. In 1846 Kiwisch introduced the vaginal warm-water douche; soon after, Cohen revived a proposition of Schweighauser, in 1825, to inject water into the cavity of the uterus. This plan is now extensively used. Guillier relates a case of sudden death following it. The author then adverted to the recent application of the caoutchouc dilater inside the os and cervix uteri, and quoted successful cases. In two cases of placenta prævia, he had thus accelerated labor, successfully opening the cervix for delivery by turning. In three cases he had induced labor by this method; one, a case of distortion of the pelvis, one of distortion with cicatrized os, and one of almost complete obliteration of the cervix by cicatricial tissue. The caoutchouc dilater acts gradually and gently; it admits of being applied to the exact spot where a dilating and exciting force is required; as a means of overcoming rigidity, it is more certain and efficient than any other. As an accelerator of labor, it is so perfect as to supersede altogether the accouchement forcé, an operation which might now be exploded. *It enables the practitioner to deliver almost at will, not only on a fixed day, but at a predetermined hour,* a power that gives us control over cases of convulsions, obstinate vomiting, exhaustion from disease or hemorrhage, much needed and not hitherto possessed. The author concluded by classifying the measures into preparatory, labor provocative, labor accelerating. We should proceed in regular gradation, prepare the soft passages, invoke contractile energy, accelerate delivery. By the judicious consecutive use of these means, it is possible to terminate a labor with certainty in a given time, and that with a greater amount of ease and security to the mother and child than has hitherto been attained. This justified the hope that the operation might be applied to the relief of emergencies and dangers which had heretofore baffled the skill of the physician.

Dr. I. Baker Brown thought the well-recorded cases of individual practitioners of greater intrinsic worth, and more reliable, than a mass of figures tabulated together to form statistical tables, often so arranged as to carry out the peculiar views of the compiler. He had for twenty-five years adopted the plan of puncturing the membranes to induce labor. Hot and cold water injections he found uncertain and tedious. The new plan was evidently an excellent one, and differed from that of Dr. Keller only in the use of water instead of air. *Incision of the os is to be highly commended,* as it is well known that in primiparous cases the os, when rigid and unyielding, is frequently lacerated by the violent expulsive uterine efforts, and yet no harm occurs to the woman from such laceration. So self-evident is this, that some gentlemen are in the habit of dividing the os with a bistoury, and, by accelerating labor, prevent the chance of laceration. The operation can be had recourse to with safety and advantage.

Dr. Priestly had found the injection of tepid water into the cervix the simplest and best, requiring only a syringe and gum catheter. The operation is almost painless, generally easily performed, and usually induces labor in less than twelve hours. One disadvantage of injecting water into the uterus, is the liability of causing a change in the position of the child, and thus producing a bad presentation. He had seen this twice.

Dr. Hall Davis thought the great object should be to save the bag of liquor amnii, as being nature's arrangement for safely dilating the passages, and defending the child from pressure. His plan was to introduce the index finger gently through the os, and upward outside the membranes as far as he could reach; he then swept it around, dislodging the mucous plug, and separating the membranes to a corresponding extent. Subsequently, a piece of oiled soft sponge was introduced, as large as could be passed without much difficulty. Where the os would not admit the fingers, he commenced with a sponge-tent, supporting it with a piece of sponge at the top of the vaginal tube. This answered in most cases. In others, he resorted to an elastic bougie, passed half way into the uterus, outside the membranes, leaving it in for five or six hours, secured by tapes. He had employed with success the injection of water into the uterus, being careful not to rupture the membranes, and guarding against the injection of air.

DR. TYLER SMITH, after the forceps, ranked premature delivery. He had tried the various methods, and found that of Kiwisch (vaginal injections) better than puncture of the membranes. For the last few years he had used uterine injections, and preferred them. The weak point of the vaginal douche was, that sometimes labor was several days, or even a week, before it came on. He injected about half a pint of warm water, of the temperature of the blood, into the uterus, through a catheter passed four or five inches into the uterus. He had never known the coming on of labor to be deferred as much as twelve hours, and generally it had commenced immediately. He had never known hemorrhage produced by it. He objected strongly against the incision of the cervix, and would regard laceration as a grave accident. He would employ the new method of Dr. Barnes, but would caution the profession against sudden dilatation, which would be liable to produce contusion, or rupture of the part.—(*Lancet*, April 13, 1861.)

VII.—*On the Removal of the Cervix Uteri.* By ROBERT GREENHALGH, M.D., London.

The safety and desirability of removal of the neck of the uterus in certain diseased conditions, is a question still *sub judice.* Though successful in the hands of Lisfranc, Hugier, and others, still fatal results have ensued, producing a very reasonable dread of the procedure. What are the most suitable cases, and by what means can the operation be most safely effected? These questions are yet to be decided.

Mrs. H., aged twenty-five, commenced to menstruate at fifteen; always suffered much pain about the back and loins at the time of the discharge, which was free and lasted four or five days. General health excellent. After marriage a "swelling came down," interfering with intercourse. This was found to be the cervix uteri protruding between the labia to the extent of an inch, enlarged, corrugated, and sensitive. A line of demarcation was found, indicating the commencement of the healthy structures. The sound passed easily into the body of the uterus to the extent of 5½ inches.

She was placed under the influence of chloroform, and about 2½ inches of the hypertrophied structure removed by the écraseur. The operation took half an hour, with very little bleeding. She progressed satisfactorily for ten days, when the catamenia appeared without pain, copious, and lasted a week. Two months later, a careful examination detected the cervix, of normal size and appearance, and the os pervious, admitting the sound to the extent of 3 inches.—(*Med. Times and Gaz.*, Sept. 7, 1861.)

VIII.—1. *Cystic Tumors of the Vagina.* By J. F. NOYES, M.D., Waterville, Mass.

Cystic tumors of the vagina are rarely met with in life. M. Nélaton describes them as having their origin in a diseased condition of the superficial and deepseated follicles of the mucous lining of the vagina. Scanzoni, however, believes they are frequently developed in the peri-vaginal cellular tissue. Rokitansky admits this view.

Mrs. S., aged forty, discovered, while pregnant, sixteen years ago, a small bunch or tumor just within and on the front of the vagina. Since her accouchement, it has rapidly increased, producing much suffering. It was found on examination to be a soft elastic tumor, as large as a coffee-cup, filling and distending the vagina, and protruding considerably from the vulva. For ten years it had occupied this position, preventing copulation, and causing dragging pains. With difficulty the finger was introduced, and it was impossible to make out how far the tumor extended up the vagina. It might easily have been taken for a prolapsus of the bladder, or an enterocele. By means of a small trocar and canula, it was found to contain a glairy substance, in color and consistence resembling thick honey. It was now fully laid open, lengthwise, and emptied, when its inner surface was found to be lined by a perfectly smooth membrane. The thickness of the cyst was nearly a quarter of an inch. The tumor was next dissected away at its base, and the edges of the wound united by silver wire. In three weeks recovery was complete.—(*Boston Med. and Surg. Journal*, April 11, 1861.)

2.—*Ibid.* By WALTER CHANNING, M.D., Boston.

Dr. Channing was called upon to attend an operation in a case, where the growth filled the vagina. It was always external when the patient was erect, occasioning great annoyance from its size, and the weight and friction of the dress. An incision was made through its whole length, and it was then carefully dissected out. No sutures were employed. The vagina was replaced, and kept *in situ* by a compress.

In another case, the tumor was bi-lobed, the whole surface red and tender. The cyst was opened freely, discharging a watery fluid, and the treatment followed out as before. These growths may be distinguished from pelvic abscess by the fact that the latter never protrudes externally, is generally in the upper part of the pelvis, and is complicated with severe constitutional symptoms, as intense pain, loss of appetite, emaciation, etc. From dislocation of the rectum and bladder they are distinguished by digital examination and the catheter.—(*Ibid.*, May 30, 1861.)

IX.—*Permanent Pressure in the Reduction of Inversio Uteri.* By M. BROCKENDAHL, Germany.

Dr. Tyler Smith has reported a case of this kind, which led M. Brockendahl to resort to the employment of continued pressure in a case brought to his notice. A primipara, aged twenty, was delivered by the forceps after a labor of twenty-four hours. Unconsciousness followed the delivery, and for several years she remained an invalid, with paralysis of the bladder, and swelling of the lower extremities, and also daily hemorrhage, in addition to regular menstruation every six weeks. After much unsuccessful treatment, a thorough examination revealed an inversion of the uterus. The womb formed in the vagina an elastic pyriform tumor $2\frac{1}{2}$ inches long, pressure upon which induced an increase of the sanguineous discharge. A thin circular fold of the vagina closely embraced the womb, and might have been mistaken for the cervix. The displacement had taken place gradually, and as no sign of peritonitis had ever presented, M. Brockendahl determined, although the inversion was complete, and the organ thick and unyielding, to make an effort at reduction. After reiterated warm baths, he attempted to insert the entire hand into the vagina, but without success, the external aperture being much contracted by a scar, the result of laceration of the perineum. Treatment was then discontinued for one year, when an attempt was made to soften the womb by kneading it with the hand, in hopes that its texture, rendered more yielding, might admit of a return of the fundus. This failing, he inserted Braun's colpeurynter, removing it daily, and again distending it to the utmost. On the fourth day, the inversion had disappeared, the cervix admitted the insertion of three fingers, and the lips were well defined. The organ was found to exceed its usual dimensions by more than half an inch. The cold douche soon restored it to its normal size, and the hemorrhage ceased entirely.—(*Deutsche Klinik; Gaz. des Hôp.*)

X.—*On Saccharine Fermentation in the Milk within the Female Breast.* By Geo. D. Gibb, M.D., London.

Dr. Gibb had a child brought to him, seven weeks old, in the most extreme emaciation, the mother appearing in perfect health. No disease could be discovered; it never seemed satisfied, but was ravenous, and required spoon food additional. The milk seemed rich, but under the microscope exhibited myriads of animalcules, *vibrio lactis*. These were evidently the result of fermentation of the saccharine element, which undoubtedly took place within the breast. There was an absence of mammary heat and congestion, but much sexual excitement, which it became necessary to control by moral and medical treatment. Cow's milk was given, and improvement rapidly ensued.

The fermentation was the result of the presence of an unusual quantity of sugar. These animalcules may be generated from the surface of the mucous membrane of the lactiferous tubes, by the fermentation of the sugar at the moment of its secretion from the blood, and hence the explanation of the large number found in milk so affected. The necessary connection existing between the mammary glands and uterine organs in the body satisfactorily explains the influence of the latter upon the former, in producing much heat and internal congestion through reflex nervous agency; these glands become morbidly irritated, as it were, and cause slight fever. This is usually early in lactation, though it has been seen in prolonged lactation, when the vision becomes impaired, and, in one case, a shade of yellow was seen by one eye, and green by the other.

In conclusion, it may be added, that when an infant becomes extremely emaciated, with copious exudations, and the mother appears healthy, with a good supply of milk, examination becomes a matter of urgent necessity; and if the milk is found to contain infusoria, it must be gradually dispensed with, and such measures adopted as shall arrest the starvation of the child.—(*Archives of Medicine.*)

